# Existence of solution and stability for the fractional order novel coronavirus (nCoV-2019) model

**DOI:** 10.1186/s13662-020-02845-0

**Published:** 2020-07-25

**Authors:** Azhar Hussain, Dumitru Baleanu, Muhammad Adeel

**Affiliations:** 1 Nonlinear Analysis Research Group, Ton Duc Thang University, Ho Chi Minh City, Vietnam; 2 Faculty of Mathematics and Statistics, Ton Duc Thang University, Ho Chi Minh City, Vietnam; 3 Department of Mathematics, University of Sargodha, Sargodha, Pakistan; 4 Department of Mathematics, Cankaya University, Ankara, Turkey; 5 Institute of Space Sciences, Magurele, Bucharest, Romania; 6 Department of Medical Research, China Medical University Hospital, China Medical University, Magurele, Taichung, Taiwan; 7 Department of Mathematics, University of Central Punjab, Lahore, Pakistan

**Keywords:** Fractional Caputo–Fabrizio derivative, Novel coronavirus (nCoV-2019), Picard–Lindelöf technique

## Abstract

The aim of this work is to present a new fractional order model of novel coronavirus (nCoV-2019) under Caputo–Fabrizio derivative. We make use of fixed point theory and Picard–Lindelöf technique to explore the existence and uniqueness of solution for the proposed model. Moreover, we explore the generalized Hyers–Ulam stability of the model using Gronwall’s inequality.

## Introduction and preliminaries

Fractional calculus plays an important role for the mathematical modeling in many scientific and engineering disciplines. For detailed study, we refer the readers to [1–14] and the references cited therein.

In the early literature, fractional derivatives in the sense of Riemann–Liouville and of Caputo were used widely. Recent studies showed that at the boundary points of the interval on which the order of derivative is based, the kernels of these derivatives have a singularity. To overcome such problems, fractional derivatives have been generalized in many other ways. For details, we refer to [15–23].

After the outbreak of novel coronavirus (nCoV-2019) on December 31, 2020, researchers started working to find the cure of the virus. Due the importance of mathematical modeling, Chen *et al.* [24] and Khan and Atangana [25] proposed the coronavirus models independently. In this paper, we generalize the novel coronavirus (nVoC-2019) model proposed by Khan and Atangana [25] by utilizing the Caputo–Fabrizio fractional derivative and explore the existence and uniqueness of its solution using fixed point theory. Also, we present the generalized Hyers–Ulam stability of it.

We now give some basic definitions which are used in the sequel.

The definition of Caputo fractional derivative can be found in many books (see, e.g., [2]).

### Definition 1

For a differentiable function *h*, the Caputo derivative of order $\gamma\in(0, 1)$ is defined by

1 $$ {}^{\mathrm{C}}\mathscr{D}^{\gamma}h(t)= \frac{1}{\varGamma(n-\gamma)} \int _{0}^{t}h{'}(s) \frac{1}{(t-s)^{\gamma}}\,ds. $$

### Definition 2

([17])

Let $h \in H^{1}(a, b)$, $a< b$, $a\in(-\infty, t)$, and $\gamma\in (0, 1)$; then the *γ*th-order Caputo–Fabrizio derivative of *h* in the Caputo sense is given as

2 $$ {}^{\mathrm{CF}}\mathscr{D}^{\gamma}h(t)= \frac{M(\gamma)}{(1-\gamma)} \int _{a}^{t}h{'}(s)\exp\biggl[- \frac{\alpha}{1-\alpha}(t-s)\biggr]\,ds, $$

where $M(\gamma)$ is a normalizing function depending on *γ* such that $M(0)=M(1)=1$.

### Definition 3

([26])

The corresponding fractional integral in the Caputo–Fabrizio sense is given by

3 $$ {}^{\mathrm{CF}}\mathscr{I}^{\gamma}h(t)= \frac{(1-\gamma)}{M(\gamma)}h(t)+\frac {\gamma}{M(\gamma)} \int_{a}^{t}h(s)\,ds,\quad 0< \gamma< 1. $$

## Fractional model in the Caputo–Fabrizio sense

Very recently, Khan and Atangana [25] proposed a mathematical model of a novel corona virus (COVID-19) as follows:

4 $$\begin{aligned}& \begin{gathered}\frac{d\mathscr{S}_{p}}{dt}=\bigwedge_{p}- \omega_{p}\mathscr {S}_{p}-\frac{\zeta_{p}\mathscr{S}_{p}(\mathscr{I}_{p}+\varPsi\mathscr {A}_{p})}{\mathscr{N}_{p}}- \omega_{w}\mathscr{S}_{p}\mathscr {M}, \\ \frac{d\mathscr{E}_{p}}{dt}=\frac{\zeta_{p}\mathscr{S}_{p}(\mathscr {I}_{p}+\varPsi\mathscr{A}_{p})}{\mathscr{N}_{p}}+\omega_{w}\mathscr {S}_{p}\mathscr{M}-(1-\varTheta_{p}) \eta_{p}\mathscr{E}_{p}-\varTheta _{p} \varrho_{p}\mathscr{E}_{p}-\varTheta_{p} \varrho_{p}\mathscr {E}_{p}-\omega_{p} \mathscr{E}_{p}, \\ \frac{d\mathscr{I}_{p}}{dt}=(1-\varTheta_{p})\eta_{p} \mathscr {E}_{p}-(\tau_{p}-\omega_{p}) \mathscr{I}_{p}, \\ \frac{d\mathscr{A}_{p}}{dt}=\varTheta_{p}\varrho_{P} \mathscr {E}_{p}-(\tau_{ap}-\omega_{p}) \mathscr{A}_{p}, \\ \frac{d\mathscr{R}_{p}}{dt}=\tau_{p}\mathscr{I}_{p}+ \tau_{ap}\mathscr {A}_{p}-\omega_{p} \mathscr{R}_{p}, \\ \frac{d\mathscr{M}}{dt}=\phi_{p}\mathscr{I}_{p}+ \varpi_{p}\mathscr {A}_{p}-\varphi\mathscr{M}, \end{gathered} \end{aligned}$$

with the initial conditions

$$\begin{aligned}& \mathscr{S}_{p}(0)=\mathscr{S}_{p}(0)\geq0,\qquad \mathscr{E}_{p}(0)=\mathscr {E}_{p}(0)\geq0,\qquad \mathscr{I}_{p}(0)=\mathscr{I}_{p}(0)\geq0, \\& \mathscr {A}_{p}(0)=\mathscr{A}_{p}(0)\geq0,\qquad \mathscr{R}_{p}(0)=\mathscr {R}_{p}(0)\geq0,\qquad \mathscr{M}(0)=\mathscr{M}(0)\geq0. \end{aligned}$$

They generalized the model to a fractional order model using Atangana–Baleanu derivative and solved the model numerically.

In this paper, we replace Atangana–Baleanu derivative with Caputo–Fabrizio fractional derivative and generalize model (4) in the following way:

5 $$\begin{aligned}& {}^{\mathrm{CF}}\mathscr{D}^{\gamma}\mathscr{S}_{p}= \bigwedge_{p}-\omega _{p} \mathscr{S}_{p}-\frac{\zeta_{p}\mathscr{S}_{p}(\mathscr {I}_{p}+\varPsi\mathscr{A}_{p})}{\mathscr{N}_{p}}-\omega_{w}\mathscr {S}_{p}\mathscr{M}, \\& \begin{gathered}{}^{\mathrm{CF}}\mathscr{D}^{\gamma}\mathscr{E}_{p}= \frac{\zeta_{p}\mathscr {S}_{p}(\mathscr{I}_{p}+\varPsi\mathscr{A}_{p})}{\mathscr{N}_{p}}+\omega _{w}\mathscr{S}_{p} \mathscr{M}-(1-\varTheta_{p})\eta_{p}\mathscr {E}_{p}-\varTheta_{p}\varrho_{p} \mathscr{E}_{p}-\varTheta_{p}\varrho _{p} \mathscr{E}_{p}-\omega_{p}\mathscr{E}_{p},\end{gathered} \\& \begin{gathered}{}^{\mathrm{CF}}\mathscr{D}^{\gamma}\mathscr{I}_{p}=(1- \varTheta_{p})\eta _{p}\mathscr{E}_{p}-( \tau_{p}-\omega_{p})\mathscr{I}_{p}, \\ {}^{\mathrm{CF}}\mathscr{D}^{\gamma}\mathscr{A}_{p}= \varTheta_{p}\varrho _{P}\mathscr{E}_{p}-( \tau_{ap}-\omega_{p})\mathscr{A}_{p},\end{gathered} \\& \begin{gathered}{}^{\mathrm{CF}}\mathscr{D}^{\gamma}\mathscr{R}_{p}= \tau_{p}\mathscr{I}_{p}+\tau _{ap} \mathscr{A}_{p}-\omega_{p}\mathscr{R}_{p}, \\ {}^{\mathrm{CF}}\mathscr{D}^{\gamma}\mathscr{M}=\phi_{p} \mathscr{I}_{p}+\varpi _{p}\mathscr{A}_{p}- \varphi\mathscr{M},\end{gathered} \end{aligned}$$

where *γ* denotes the fractional order parameter and the model variables in (4) are nonnegative, the initial conditions are given by

$$\begin{aligned}& \mathscr{S}_{p}(0)=\mathscr{S}_{p}(0)\geq0,\qquad \mathscr{E}_{p}(0)=\mathscr {E}_{p}(0)\geq0,\qquad \mathscr{I}_{p}(0)=\mathscr{I}_{p}(0)\geq0, \\& \mathscr {A}_{p}(0)=\mathscr{A}_{p}(0)\geq0,\qquad \mathscr{R}_{p}(0)=\mathscr {R}_{p}(0)\geq0,\qquad \mathscr{M}(0)=\mathscr{M}(0)\geq0. \end{aligned}$$

Using the initial conditions and fractional integral operator, we convert model (5) into the following integral equations:

6 $$\begin{aligned}& \begin{gathered}\mathscr{S}_{p}(t)-\mathscr{S}_{p}(0)={}^{\mathrm{CF}} \mathscr{I}^{\gamma} \biggl[\bigwedge_{p}- \omega_{p}\mathscr{S}_{p}-\frac{\zeta_{p}\mathscr {S}_{p}(\mathscr{I}_{p}+\varPsi\mathscr{A}_{p})}{\mathscr{N}_{p}}-\omega _{w}\mathscr{S}_{p}\mathscr{M} \biggr], \\ \mathscr{E}_{p}(t)-\mathscr{E}_{p}(0)={}^{\mathrm{CF}} \mathscr{I}^{\gamma} \biggl[\frac{\zeta_{p}\mathscr{S}_{p}(\mathscr{I}_{p}+\varPsi\mathscr {A}_{p})}{\mathscr{N}_{p}}+\omega_{w} \mathscr{S}_{p}\mathscr {M}-(1-\varTheta_{p}) \eta_{p}\mathscr{E}_{p} \\ \phantom{\mathscr{E}_{p}(t)-\mathscr{E}_{p}(0)={}}-\varTheta_{p}\varrho_{p}\mathscr{E}_{p}- \varTheta_{p}\varrho _{p}\mathscr{E}_{p}- \omega_{p}\mathscr{E}_{p} \biggr], \\ \mathscr{I}_{p}(t)-\mathscr{I}_{p}(0)={}^{\mathrm{CF}} \mathscr{I}^{\gamma }\bigl[(1-\varTheta_{p}) \eta_{p}\mathscr{E}_{p}-(\tau_{p}- \omega_{p})\mathscr {I}_{p}\bigr], \\ \mathscr{A}_{p}(t)-\mathscr{A}_{p}(0)={}^{\mathrm{CF}} \mathscr{I}^{\gamma }\bigl[\varTheta_{p}\varrho_{P} \mathscr{E}_{p}-(\tau_{ap}-\omega_{p}) \mathscr {A}_{p}\bigr], \\ \mathscr{R}_{p}(t)-\mathscr{R}_{p}(0)={}^{\mathrm{CF}} \mathscr{I}^{\gamma}[\tau _{p}\mathscr{I}_{p}+ \tau_{ap}\mathscr{A}_{p}-\omega_{p}\mathscr {R}_{p}], \\ \mathscr{M}(t)-\mathscr{M}(0)={}^{\mathrm{CF}}\mathscr{I}^{\gamma}[\phi _{p}\mathscr{I}_{p}+\varpi_{p} \mathscr{A}_{p}-\varphi\mathscr {M}].\end{gathered} \end{aligned}$$

For the sake of convenience, we assume the kernels

7 $$\begin{aligned}& \begin{gathered}\mathscr{K}_{1}(t,\mathscr{S}_{p})= \bigwedge_{p}-\omega_{p}\mathscr {S}_{p}(t)-\frac{\zeta_{p}\mathscr{S}_{p}(t)(\mathscr{I}_{p}(t)+\varPsi \mathscr{A}_{p}(t))}{\mathscr{N}_{p}(t)}-\omega_{w}\mathscr {S}_{p}(t)\mathscr{M}(t), \\ \mathscr{K}_{2}(t,\mathscr{E}_{p})= \frac{\zeta_{p}\mathscr {S}_{p}(t)(\mathscr{I}_{p}(t)+\varPsi\mathscr{A}_{p}(t))}{\mathscr {N}_{p}(t)}+\omega_{w}\mathscr{S}_{p}(t) \mathscr{M}(t)-(1-\varTheta _{p})\eta_{p} \mathscr{E}_{p}(t)-\varTheta_{p}\varrho_{p} \mathscr {E}_{p}(t)\hspace{-12pt} \\ \phantom{\mathscr{K}_{2}(t,\mathscr{E}_{p})=} {}-\varTheta_{p}\varrho_{p}\mathscr {E}_{p}(t)-\omega_{p}\mathscr{E}_{p}(t), \\ \mathscr{K}_{3}(t,\mathscr{I}_{p})=(1- \varTheta_{p})\eta_{p}\mathscr {E}_{p}(t)-( \tau_{p}-\omega_{p})\mathscr{I}_{p}(t), \\ \mathscr{K}_{4}(t,\mathscr{A}_{p})= \varTheta_{p}\varrho_{P}\mathscr {E}_{p}(t)-( \tau_{ap}-\omega_{p})\mathscr{A}_{p}(t), \\ \mathscr{K}_{5}(t,\mathscr{R}_{p})= \tau_{p}\mathscr{I}_{p}(t)+\tau _{ap} \mathscr{A}_{p}(t)-\omega_{p}\mathscr{R}_{p}(t), \\ \mathscr{K}_{6}(t,\mathscr{M})=\phi_{p} \mathscr{I}_{p}(t)+\varpi _{p}\mathscr{A}_{p}(t)- \varphi\mathscr{M}(t)\end{gathered} \end{aligned}$$

and the functions

8 $$ \varUpsilon(\gamma)=\frac{1-\gamma}{M(\gamma)},\qquad \varPhi(\gamma)= \frac {\gamma}{M(\gamma)}. $$

Using (3), (7), and (8) in (6) and writing state variables in terms of kernels, we obtain

9 $$\begin{aligned}& \begin{gathered} \mathscr{S}_{p}(t)=\mathscr{S}_{p}(0)+ \varUpsilon(\gamma)\mathscr {K}_{1}(t,\mathscr{S}_{p})+ \varPhi(\gamma) \int_{a}^{t}\mathscr {K}_{1}(x, \mathscr{S}_{p})\,dx, \\ \mathscr{E}_{p}(t)=\mathscr{E}_{p}(0)+\varUpsilon( \gamma)\mathscr {K}_{2}(t,\mathscr{E}_{p})+\varPhi( \gamma) \int_{a}^{t}\mathscr {K}_{2}(x, \mathscr{E}_{p})\,dx, \\ \mathscr{I}_{p}(t)=\mathscr{I}_{p}(0)+\varUpsilon( \gamma)\mathscr {K}_{3}(t,\mathscr{I}_{p})+\varPhi( \gamma) \int_{a}^{t}\mathscr {K}_{3}(x, \mathscr{I}_{p})\,dx, \\ \mathscr{A}_{p}(t)=\mathscr{A}_{p}(0)+\varUpsilon( \gamma)\mathscr {K}_{4}(t,\mathscr{A}_{p})+\varPhi( \gamma) \int_{a}^{t}\mathscr {K}_{4}(x, \mathscr{A}_{p})\,dx, \\ \mathscr{R}_{p}(t)=\mathscr{R}_{p}(0)+\varUpsilon( \gamma)\mathscr {K}_{5}(t,\mathscr{R}_{p})+\varPhi( \gamma) \int_{a}^{t}\mathscr {K}_{5}(x, \mathscr{R}_{p})\,dx, \\ \mathscr{M}(t)=\mathscr{M}(0)+\varUpsilon(\gamma)\mathscr {K}_{6}(t, \mathscr{M})+\varPhi(\gamma) \int_{a}^{t}\mathscr {K}_{6}(x, \mathscr{M})\,dx.\end{gathered} \end{aligned}$$

The Picard iterations are given by

10 $$\begin{aligned}& \begin{gathered}\mathscr{S}_{p}^{j+1}(t)=\varUpsilon( \gamma)\mathscr{K}_{1}\bigl(t,\mathscr {S}_{p}^{j} \bigr)+\varPhi(\gamma_{1}) \int_{a}^{t}\mathscr{K}_{1}\bigl(x, \mathscr {S}^{j}_{p}\bigr)\,dx, \\ \mathscr{E}_{p}^{j+1}(t)=\varUpsilon( \gamma_{2})\mathscr {K}_{2}\bigl(t,\mathscr{E}_{p}^{j} \bigr)+\varPhi(\gamma_{2}) \int_{a}^{t}\mathscr {K}_{2} \bigl(x,\mathscr{E}_{p}^{j}\bigr)\,dx, \\ \mathscr{I}_{p}^{j+1}(t)=\varUpsilon( \gamma_{3})\mathscr {K}_{3}\bigl(t,\mathscr{I}_{p}^{j} \bigr)+\varPhi(\gamma_{3}) \int_{a}^{t}\mathscr {K}_{3} \bigl(x,\mathscr{I}_{p}^{j}\bigr)\,dx, \\ \mathscr{A}_{p}^{j+1}(t)=\varUpsilon( \gamma_{4})\mathscr {K}_{4}\bigl(t,\mathscr{A}_{p}^{j} \bigr)+\varPhi(\gamma_{4}) \int_{a}^{t}\mathscr {K}_{4} \bigl(x,\mathscr{A}_{p}^{j}\bigr)\,dx, \\ \mathscr{R}_{p}^{j+1}(t)=\varUpsilon( \gamma_{5})\mathscr {K}_{5}\bigl(t,\mathscr{R}_{p}^{j} \bigr)+\varPhi(\gamma_{5}) \int_{a}^{t}\mathscr {K}_{5} \bigl(x,\mathscr{R}_{p}^{j}\bigr)\,dx, \\ \mathscr{M}^{j+1}(t)=\varUpsilon(\gamma_{6}) \mathscr{K}_{6}\bigl(t,\mathscr {M}^{j}\bigr)+\varPhi( \gamma_{6}) \int_{a}^{t}\mathscr{K}_{6}\bigl(x, \mathscr {M}^{j}\bigr)\,dx.\end{gathered} \end{aligned}$$

In order to show the existence and uniqueness of solution of model (5), we make use of fixed point theory and Picard–Lindelöf technique. First, we re-write model (5) in the following way:

11 $$ \textstyle\begin{cases} {}^{\mathrm{CF}}\mathscr{D}^{\gamma}\psi(t)=\mathscr{K}(t, \psi(t)), \\ \psi(0)=\psi_{0},\quad 0< t< T< \infty. \end{cases} $$

The vector $\psi(t)=(\mathscr{S}_{p}, \mathscr{E}_{p}, \mathscr{I}_{p}, \mathscr{A}_{p}, \mathscr{R}_{p}, \mathscr{M})$ and $\mathscr{K}$ in (10) represent the state variables and a continuous vector function respectively defined as follows:

12 $$ \begin{aligned}[b]\mathscr{K}&=\left ( \textstyle\begin{array}{c} \mathscr{K}_{1} \\ \mathscr{K}_{2} \\ \mathscr{K}_{3} \\ \mathscr{K}_{4} \\ \mathscr{K}_{6} \\ \mathscr{K}_{6} \end{array}\displaystyle \right )\\&=\left ( \textstyle\begin{array}{c} \bigwedge_{p}-\omega_{p}\mathscr{S}_{p}(t)-\frac{\zeta_{p}\mathscr {S}_{p}(t)(\mathscr{I}_{p}(t)+\varPsi\mathscr{A}_{p}(t))}{\mathscr {N}_{p}(t)}-\omega_{w}\mathscr{S}_{p}(t)\mathscr{M}(t) \\ \frac{\zeta_{p}\mathscr{S}_{p}(t)(\mathscr{I}_{p}(t)+\varPsi\mathscr {A}_{p}(t))}{\mathscr{N}_{p}(t)}+\omega_{w}\mathscr{S}_{p}(t)\mathscr {M}(t)-(1-\varTheta_{p})\eta_{p}\mathscr{E}_{p}(t)-\varTheta_{p}\varrho _{p}\mathscr{E}_{p}(t) \\ -\varTheta_{p}\varrho_{p}\mathscr {E}_{p}(t)-\omega_{p}\mathscr{E}_{p}(t) \\ (1-\varTheta_{p})\eta_{p}\mathscr{E}_{p}(t)-(\tau_{p}-\omega _{p})\mathscr{I}_{p}(t) \\ \varTheta_{p}\varrho_{P}\mathscr{E}_{p}(t)-(\tau_{ap}-\omega _{p})\mathscr{A}_{p}(t) \\ \tau_{p}\mathscr{I}_{p}(t)+\tau_{ap}\mathscr{A}_{p}(t)-\omega _{p}\mathscr{R}_{p}(t) \\ \phi_{p}\mathscr{I}_{p}(t)+\varpi_{p}\mathscr{A}_{p}(t)-\varphi\mathscr{M}(t) \end{array}\displaystyle \right )\end{aligned} $$

with the initial conditions $\psi_{0}(t)=(\mathscr{S}_{p}(0), \mathscr {E}_{p}(0), \mathscr{I}_{p}(0), \mathscr{A}_{p}(0), \mathscr{R}_{p}(0), \mathscr{M}(0))$. Corresponding to (11), the integral equation is given by

13 $$ \psi(t)=\psi_{0}+\varUpsilon(\gamma)\mathscr{K} \bigl(t,\psi(t)\bigr)+\varPhi (\gamma) \int_{a}^{t}\mathscr{K}\bigl(x,\psi(x)\bigr)\,dx. $$

Moreover, $\mathscr{K}$ satisfies the Lipschitz condition given by

14 $$ \bigl\Vert \mathscr{K}\bigl(t, \psi_{1}(t)\bigr)- \mathscr{K}\bigl(t, \psi_{2}(t)\bigr) \bigr\Vert \leq \varOmega \bigl\Vert \psi_{1}(t)-\psi_{2}(t) \bigr\Vert . $$

### Theorem 1

*Assuming* (14), *there exists a unique solution of* (11) *if*

15 $$ \bigl(\varUpsilon(\gamma)+T\varPhi(\gamma)\bigr)\varOmega< 1. $$

### Proof

Consider $A=[0, T]$, $\mathcal{X}=\mathcal{C}(A, \mathbb{R}^{6})$ and the Picard operator $\mathcal{T}:\mathcal{X}\to\mathcal{X}$ defined by

16 $$ \mathcal{T}\bigl[\psi(t)\bigr]=\psi_{0}+ \varUpsilon(\gamma)\mathscr{K}\bigl(t,\psi (t)\bigr)+\varPhi(\gamma) \int_{0}^{t}\mathscr{K}\bigl(x,\psi(x)\bigr)\,dx, $$

which turns equation (13) to

17 $$ \psi(t)=\mathcal{T}\bigl[\psi(t)\bigr]. $$

Together with the supremum norm $\|\cdot\|_{A}$ on *ψ* given by

18 $$ \bigl\Vert \psi(t) \bigr\Vert _{A}=\sup _{t\in A} \bigl\Vert \psi(t) \bigr\Vert ,\quad \psi(t)\in \mathcal{X}, $$

$\mathcal{X}$ defines a Banach space.

It is to be noted that the solution of the fractional order novel coronavirus (nCoV-2019) model is bounded, i.e.,

$$\begin{aligned} \bigl\Vert \mathcal{T}\bigl[\psi(t)\bigr]-\psi_{0} \bigr\Vert _{A} =& \biggl\Vert \varUpsilon(\gamma ) \bigl(\mathscr{K}\bigl(t, \psi(t)\bigr)\bigr) + \varPhi(\gamma) \int_{0}^{t}\mathscr {K}\bigl(x,\psi(x)\bigr)\,dx \biggr\Vert _{A} \\ \leq& \varUpsilon(\gamma) \bigl\Vert \mathscr{K}\bigl(t,\psi(t)\bigr) \bigr\Vert _{A} + \varPhi (\gamma) \int_{0}^{t} \bigl\Vert \mathscr{K}\bigl(x, \psi(x)\bigr) \bigr\Vert _{A}\,dx \\ \leq& \bigl(\varUpsilon(\gamma)+T\varPhi(\gamma)\bigr)\varOmega< 1. \end{aligned}$$

Now using Picard operator equation (16), we have

$$\begin{aligned}& \bigl\Vert \mathcal{T}\bigl[\psi_{1}(t)\bigr]-\mathcal{T}\bigl[ \psi_{2}(t)\bigr] \bigr\Vert _{A} \\& \quad= \biggl\Vert \varUpsilon(\gamma) \bigl(\mathscr{K}\bigl(t, \psi_{1}(t)\bigr)-\mathscr {K}\bigl(t,\psi_{2}(t)\bigr) \bigr) + \varPhi(\gamma) \int_{0}^{t}\bigl(\mathscr{K}\bigl(x,\psi _{1}(x)\bigr)-\mathscr{K}\bigl(x,\psi_{1}(x)\bigr) \bigr)\,dx \biggr\Vert _{A} \\& \quad\leq \varUpsilon(\gamma) \bigl\Vert \mathscr{K}\bigl(t,\psi_{1}(t) \bigr)-\mathscr {K}\bigl(t,\psi_{2}(t)\bigr) \bigr\Vert _{A} + \varPhi(\gamma) \int_{0}^{t} \bigl\Vert \mathscr {K}\bigl(x, \psi_{1}(x)\bigr)-\mathscr{K}\bigl(x,\psi_{1}(x)\bigr) \bigr\Vert _{A}\,dx \\& \quad\leq \varUpsilon(\gamma)\varOmega \bigl\Vert \psi_{1}(t)- \psi_{2}(t) \bigr\Vert _{A} + \varPhi(\gamma)\varOmega \int_{0}^{t} \bigl\Vert \psi_{1}(x)- \psi_{2}(x) \bigr\Vert _{A}\,dx \\& \quad\leq \bigl(\varUpsilon(\gamma)+T\varPhi(\gamma)\bigr)\varOmega \bigl\Vert \psi _{1}(t)-\psi_{2}(t) \bigr\Vert _{A} \\& \quad= \mathcal{A} \bigl\Vert \psi_{1}(t)-\psi_{2}(t) \bigr\Vert _{A}, \end{aligned}$$

where

$$ \mathcal{A}=\bigl(\varUpsilon(\gamma)+T\varPhi(\gamma)\bigr)\varOmega. $$

This implies

19 $$ \bigl\Vert \mathcal{T}\bigl[\psi_{1}(t)\bigr]-\mathcal{T}\bigl[ \psi_{2}(t)\bigr] \bigr\Vert _{A}\leq\mathcal {A} \bigl\Vert \psi_{1}(t)-\psi_{2}(t) \bigr\Vert _{A}. $$

Thus the defined operator $\mathcal{T}$ is a contraction, and hence model (11) has a unique solution. □

### Remark 1

We remark here that the stability by considering disease free equilibrium and the endemic equilibrium for model (11) can be proved on the same lines as given in [25].

## Generalized Hyers–Ulam stability

In this section, we explore the stability analysis of model (11).

### Definition 4

Let $0<\gamma<1$ and $\mathscr{K}: [0, T]\times\mathbb{R}^{6}\to \mathbb{R}^{6}$ be a continuous function. Then (11) is Hyers–Ulam stable if there exist $L>0$ and $\epsilon>0$ such that, for each solution $\psi\in\mathcal{C}([0, T], \mathbb{R}^{6})$ of

20 $$ \bigl\vert {}^{\mathrm{CF}}\mathscr{D}^{\gamma}\psi(t)-\mathscr{K}\bigl(t, \psi(t)\bigr) \bigr\vert \leq \epsilon\quad \forall~ t \in[0, T], $$

there exists a solution $\psi' \in\mathcal{C}([0, T], \mathbb{R}^{6})$ of (11) with

21 $$ \bigl\vert \psi(t)-\psi'(t) \bigr\vert \leq L\epsilon \quad\forall ~t \in[0, T]. $$

### Definition 5

Let $0<\gamma<1$ and $\mathscr{K}: [0, T]\times\mathbb{R}^{6}\to \mathbb{R}^{6}$ and $\varPi:[0, T]\to\mathbb{R_{+}}$ be a continuous function. Then (11) is generalized Hyers–Ulam–Rassias stable with respect to *Π* if there exists a constant $C_{\mathscr{K}, \varPi}>0$ such that, for each solution $\psi\in\mathcal{C}([0, T], \mathbb{R}^{6})$ of

22 $$ \bigl\vert {}^{\mathrm{CF}}\mathscr{D}^{\gamma}\psi(t)- \mathscr{K}\bigl(t, \psi(t)\bigr) \bigr\vert \leq\varPi (t) \quad\forall ~t \in[0, T], $$

there exists a solution $\psi' \in\mathcal{C}([0, T], \mathbb{R}^{6})$ of (11) with

23 $$ \bigl\vert \psi(t)-\psi'(t) \bigr\vert \leq C_{\mathscr{K}, \varPi}\varPi(t) \quad\forall ~t \in [0, T]. $$

Assume the following:

[$\mathcal{A}_{1}$]
$\mathscr{K}: [0, T]\times\mathbb{R}^{6}\to \mathbb{R}^{6}$ is continuous;
[$\mathcal{A}_{2}$]
there exists $C_{\mathscr{K}}>0$ such that
  $$\bigl\vert \mathscr{K}(t, \psi)-\mathscr{K}\bigl(x, \psi'\bigr) \bigr\vert \leq C_{\mathscr{K}} \bigl\vert \psi -\psi' \bigr\vert $$
  for all $\psi, \psi' \in\mathbb{R}^{6}$, $t\in[0, T]$;
[$\mathcal{A}_{3}$]
let $\varPi\in\mathcal{C}([0, T], \mathbb {R}_{+})$ be an increasing function, and let there exist $\lambda _{\varPi}>0$ such that
  24 $$ \int_{0}^{t}\varPi(x)\,dx \leq \lambda_{\varPi}\varPi(t) $$
  for all $x\in[0, T]$.

### Theorem 2

*Assuming* [$\mathcal{A}_{1}$]*–*[$\mathcal{A}_{3}$] *hold*, (11) *is generalized Ulam–Hyers–Rassias stable with respect toΠon*$[0, T]$*provided that*$\varUpsilon(\gamma)\mathcal{C}_{\mathscr{K}}<1$.

### Proof

Let $\psi'\in\mathcal{C}([0, T], \mathbb{R}^{6})$ be a solution of (11). Then, from Theorem 1, model (11) has the unique solution

25 $$ \psi(t)=\psi_{0}+\varUpsilon(\gamma)\mathscr{K} \bigl(t,\psi(t)\bigr)+\varPhi (\gamma) \int_{0}^{t}\mathscr{K}\bigl(x, \psi(x)\bigr)\,dx,\quad t\in[0, T]. $$

From (22), we have

$$\begin{aligned}& \biggl\vert \psi'(t)-\psi_{0}+\varUpsilon(\gamma)\mathscr{K} \bigl(t,\psi '(t)\bigr)+\varPhi(\gamma) \int_{0}^{t}\mathscr{K}\bigl(x, \psi'(x)\bigr)\,dx \biggr\vert \\& \quad\leq \varUpsilon(\gamma)\varPi(t) + \varPhi(\gamma) \int_{0}^{t}\varPi (x)\,dx \\& \quad\leq \bigl(\varUpsilon(\gamma)+\varPhi(\gamma)\lambda_{\varPi}\bigr) \varPi(t). \end{aligned}$$

Thus

$$\begin{aligned}& \begin{gathered} \bigl\vert \psi'(t)-\psi(t) \bigr\vert \\ \quad\leq \biggl\vert \psi'(t)-\psi_{0}-\varUpsilon(\gamma) \mathscr{K}\bigl(t,\psi(t)\bigr)-\varPhi(\gamma) \int_{0}^{t}\mathscr{K}\bigl(x, \psi(x)\bigr)\,dx \biggr\vert \\ \quad\leq \biggl\vert \psi'(t)-\psi_{0}-\varUpsilon(\gamma) \mathscr{K}\bigl(t,\psi '(t)\bigr)- \varPhi(\gamma) \int_{0}^{t}\mathscr{K}\bigl(x, \psi'(x)\bigr)\,dx - \varUpsilon(\gamma)\mathscr{K}\bigl(t,\psi(t) \bigr) \\ \qquad{}-\varPhi(\gamma) \int_{0}^{t}\mathscr{K}\bigl(x,\psi(x)\bigr)\,dx + \varUpsilon (\gamma)\mathscr{K}\bigl(t,\psi'(t)\bigr)+\varPhi(\gamma) \int_{0}^{t}\mathscr {K}\bigl(x,\psi'(x)\bigr)\,dx \biggr\vert \\ \quad\leq \biggl\vert \psi'(t)-\psi_{0}-\varUpsilon(\gamma) \mathscr{K}\bigl(t,\psi'(t)\bigr)-\varPhi(\gamma) \int_{0}^{t}\mathscr{K}\bigl(x,\psi'(x)\bigr)\,dx \biggr\vert \\\qquad{}+ \varUpsilon(\gamma) \bigl\vert \mathscr{K}\bigl(t,\psi(t)\bigr)- \mathscr{K}\bigl(t,\psi'(t)\bigr) \bigr\vert \\ \qquad{}+\varPhi(\gamma) \int_{0}^{t} \bigl\vert \mathscr{K}\bigl(x, \psi(x)\bigr)- \mathscr {K}\bigl(x,\psi'(x)\bigr) \bigr\vert \, dx \\ \quad\leq \bigl(\varUpsilon(\gamma)+\varPhi(\gamma)\lambda_{\varPi}\bigr) \varPi(t) + \varUpsilon(\gamma)C_{\mathscr{K}} \bigl\vert \psi(t)- \psi'(t) \bigr\vert +\varPhi(\gamma )C_{\mathscr{K}} \int_{0}^{t} \bigl\vert \psi(x)- \psi'(x) \bigr\vert \, dx.\end{gathered} \end{aligned}$$

Now, $\varUpsilon(\gamma)C_{\mathscr{K}}<1$, so

26 $$ \bigl\vert \psi(t)-\psi'(t) \bigr\vert \leq \frac{(\varUpsilon(\gamma)+\varPhi(\gamma)\lambda _{\varPi})\varPi(t)}{1-\varUpsilon(\gamma)}+\frac{\varPhi(\gamma )C_{\mathscr{K}}}{1-\varUpsilon(\gamma)} \int_{0}^{t} \bigl\vert \psi(x)- \psi'(x) \bigr\vert \,dx. $$

From Gronwall’s inequality, we have

$$\bigl\vert \psi(t)-\psi'(t) \bigr\vert \leq \biggl[ \frac{\varUpsilon(\gamma)+\varPhi(\gamma )\lambda_{\varPi}}{1-\varUpsilon(\gamma)}\exp(t) \biggr]\varPi(t). $$

Setting $C_{\mathscr{K}, \varPi}= [\frac{\varUpsilon(\gamma )+\varPhi(\gamma)\lambda_{\varPi}}{1-\varUpsilon(\gamma )}\exp(t) ]$, we arrived at

$$\bigl\vert \psi(t)-\psi'(t) \bigr\vert \leq C_{\mathscr{K},\varPi}\varPi(t). $$

This completes the proof. □

## Conclusion

In this paper, we discussed the novel corona virus model given in [25] within the Caputo–Fabrizio fractional model, and we showed the existence and uniqueness of its solution by applying the Banach contraction principle and Picard–Lindelöf technique. Utilizing Gronwall’s inequality, we presented the generalized Hyers–Ulam stability of the fractional model.
